# Emergency room comprehensive assessment of demographic, radiological, laboratory and clinical data of patients with COVID-19: determination of its prognostic value for in-hospital mortality

**DOI:** 10.1007/s11739-021-02669-0

**Published:** 2021-03-08

**Authors:** Marco Gatti, Marco Calandri, Andrea Biondo, Carlotta Geninatti, Clara Piatti, Irene Ruggirello, Ambra Santonocito, Sara Varello, Laura Bergamasco, Paolo Bironzo, Adriana Boccuzzi, Luca Brazzi, Pietro Caironi, Luciano Cardinale, Rossana Cavallo, Franco Riccardini, Giorgio Limerutti, Andrea Veltri, Paolo Fonio, Riccardo Faletti

**Affiliations:** 1 Department of Surgical Sciences, Radiology Unit, University of Turin, Turin, Italy; 2 Radiology Department A.O.U. San Luigi Gonzaga, Regione Gonzole 10, Orbassano, Italy; 3 Department of Oncology, University of Turin, Turin, Italy; 4 Department of Surgical Sciences, University of Turin, Turin, Italy; 5 Thoracic Oncology Unit, A.O.U. San Luigi Gonzaga, Regione Gonzole 10, Orbassano, Italy; 6 Emergency Department, San Luigi Gonzaga University Hospital, Orbassano, TO Italy; 7 Department of Surgical Sciences, Anesthesia Unit, University of Turin, Turin, Italy; 8 Department of Anesthesia and Critical Care, A.O.U. San Luigi Gonzaga, Regione Gonzole 10, Orbassano, Italy; 9 Department of Public Health and Pediatrics, Laboratory of Microbiology and Virology, Città della Salute e della Scienza Hospital, University of Turin, Turin, Italy; 10 Department of Medical Science, University of Turin, Turin, Italy; 11 Department of Radiology, S.C. Radiodiagnostica Ospedaliera, Turin, Italy

**Keywords:** Chest X-ray, Coronavirus disease 2019 (COVID-19), Severe acute respiratory syndrome coronavirus 2 (SARS-CoV-2), Prognosis, Outcome

## Abstract

Mortality risk in COVID-19 patients is determined by several factors. The aim of our study was to adopt an integrated approach based on clinical, laboratory and chest x-ray (CXR) findings collected at the patient’s admission to Emergency Room (ER) to identify prognostic factors. Retrospective study on 346 consecutive patients admitted to the ER of two North-Western Italy hospitals between March 9 and April 10, 2020 with clinical suspicion of COVID-19 confirmed by reverse transcriptase-polymerase reaction chain test (RT-PCR), CXR performed within 24 h (analyzed with two different scores) and recorded prognosis. Clinical and laboratory data were collected. Statistical analysis on the features of 83 in-hospital dead vs 263 recovered patients was performed with univariate (uBLR), multivariate binary logistic regression (mBLR) and ROC curve analysis. uBLR identified significant differences for several variables, most of them intertwined by multiple correlations. mBLR recognized as significant independent predictors for in-hospital mortality age > 75 years, C-reactive protein (CRP) > 60 mg/L, PaO_2_/FiO_2_ ratio (P/F) < 250 and CXR “Brixia score” > 7. Among the patients with at least two predictors, the in-hospital mortality rate was 58% against 6% for others [*p* < 0.0001; RR = 7.6 (4.4–13)]. Patients over 75 years had three other predictors in 35% cases against 10% for others [*p* < 0.0001, RR = 3.5 (1.9–6.4)]. The greatest risk of death from COVID-19 was age above 75 years, worsened by elevated CRP and CXR score and reduced P/F. Prompt determination of these data at admission to the emergency department could improve COVID-19 pretreatment risk stratification.

## Introduction

Coronavirus disease 2019 (COVID-19) is caused by Severe Acute Respiratory Syndrome Coronavirus 2 (SARS-CoV-2). Medical symptoms vary from asymptomatic inflammation to a wide range of systemic and/or respiratory manifestations, causing a mortality rate of about 2.4% according to the World Health Organization (WHO) [1]. Recent data suggest that the mortality risk is higher in patients with advanced age, presence of systemic comorbidities, inflammatory markers and extent of lung anomalies [2–5]. An early and comprehensive assessment of all these parameters is therefore, crucial as soon as a suspected COVID-19 patient arrives to the emergency room.

Imaging plays a key role in the evaluation of COVID-19 patients by assessing the extent of pulmonary involvement. Even if computed tomography (CT) is the technique with the highest sensitivity for the evaluation of the patient's prognosis [6–9], the Fleischner Society's Multinational Consensus Statement [10] stated that the CT scan should not be used for screening or as a first-line diagnostic test for COVID-19, also because the use of a non-dedicated CT scanner requires time-consuming and laborious decontamination procedures to reduce the risk of cross-infection. Chest x-ray (CXR) suffers from low sensitivity [11]; on the other hand, it has the advantage of the ease and speed of equipment cleaning and the wide availability of portable devices. Therefore, many radiological societies [12, 13] recommend the use of CXR as a first-line imaging tool in emergency departments, reserving specific roles for chest CT, as identifying typical features of COVID-19 pneumonia in selected cases. In this perspective, a few studies have evaluated the contribution of CXR to the prognostic assessment of patients with COVID-19 [14–16], confirming that extensive lung involvement is associated with several demographic and clinical characteristics of the patients leading to a worse prognosis.

Even if the evaluation of a patient admitted to an emergency room includes a variety of clinical, laboratory and CXR findings, to the best of our knowledge there are no studies on how the entire set of these data and their relations impact on the early COVID-19 patient’s prognostic assessment. We considered the issue worth of further exploration and planned a retrospective study based on an integrated approach to the data usually collected in ER to better define the comprehensive scenario behind the positive or negative outcome of COVID-19 patients.

## Methods

### Study design and population

The study, piloted in agreement with the 1964 Helsinki declaration and its later amendments, was approved by the institutional review board. The requirement for informed patient consent was waived.

**Fig. 1:**
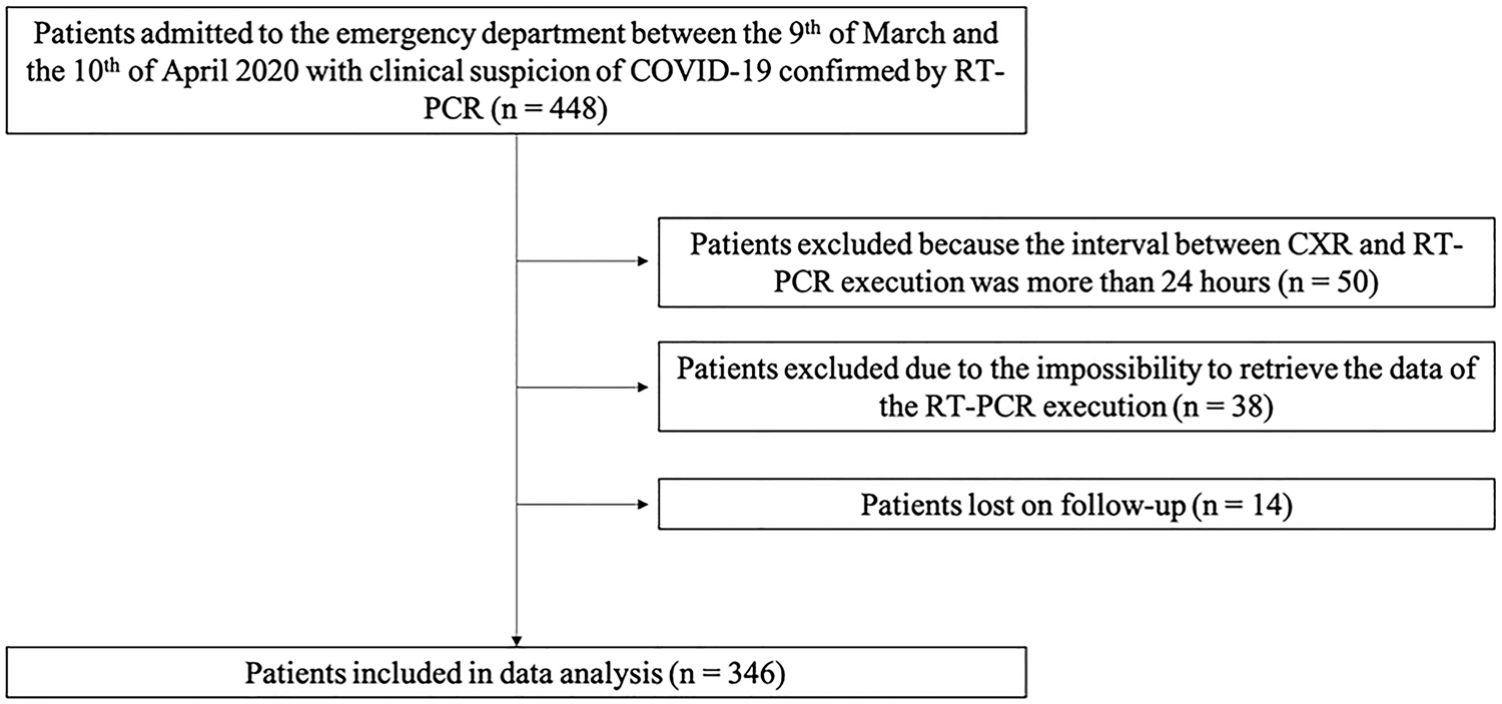
Study flow chart

This was a retrospective two-center study on 448 consecutive patients admitted to the emergency department of two large North-Western Italy hospitals between March 9 and April 10, 2020, at the peak of the local Covid-19 pandemic outburst. The study flow chart is reported in Fig. 1. The final study sample was composed of 346 patients who met the inclusion criteria of: (1) clinical suspicion of COVID-19 confirmed by RT-PCR, (2) CXR performed within 24 h of the swab execution, (3) official records of the prognosis.

The primary outcome variable was in-hospital mortality; primary predictors was the ER data on demographical, clinical and laboratory parameters and CXR outcome.

### Clinical and laboratory data

The clinical and laboratory data of the patients were collected in accordance with the structured report released by the “Società Italiana di Radiologia Medica e Interventistica (SIRM)” [17]. Comorbidities included in the analysis were: presence of diabetes, obesity (i.e. BMI > 30), hypertension, smoke history, ACEi/Sartan or FANS therapy. As for clinical data: fever, cough, rhinitis, dyspnea, anosmia, dysgeusia, pharyngodynia, myalgias, asthenia, conjunctivitis, headache, nausea, vomit and diarrhea. Regarding laboratory data: white blood cells (WBC) count (neutrophils, lymphocytes), procalcitonin (PCT), C-reactive protein (CRP), lactate dehydrogenase (LDH), hepatic enzymes, creatine kinase (CK), blood pH and arterial partial pressure of oxygen (PaO_2_) and carbon dioxide (PaCO_2_) and fraction of inspired oxygen (FiO_2_). Moreover, we calculated the neutrophil–to-lymphocyte ratio (NLR), the alveolar–arterial gradient (A–a gradient) and the PaO_2_/FiO_2_ ratio (P/F). The RT-PCR protocol included extraction with QIAsymphony^®^ DSP Virus/Pathogen Midi Kit and amplification with Seegene AllplexTM 2019-nCoV Assay (target genes E, N, RdRP).

### Image acquisition and analysis

CXRs were acquired as computerized or digital radiographs following the local protocols. All CXR studies were analyzed by two observers (MC and MG with more than 5 years’ experience) using a picture archiving and communication system (PACS) workstation [Carestream Vue PACS v11.3.4 (Carestream Health, Inc, Rochester, NY)]. In the few cases of disagreement, the decision was reached by consultation with a senior radiologist (RF with more than 10 years’ experience).

CXRs were evaluated using two separate scores: the first [14] measured the involvement of each lung field (upper, middle and lower) in a binary manner, ranging from 0 to 6; whereas, the second, the so-called “Brixia score” [18], assessed the severity of the involvement of each lung field on a scale from 0 to 3, for a total score from 0 to 18. The presence of pleural effusion and reduction of lung volumes were also evaluated.

### Statistical analysis

The first step of the statistical analysis of the set of data included univariate tests. Continuous variables were checked for normality with the Shapiro–Wilks *W* test. Age was normal and was thus expressed as mean and standard deviation. Normality was rejected for all other data, which were thus expressed as median with first (Q1) and third (Q3) quartile; categorical variables were presented as absolute numbers and percentages. Univariate binary logistic regression (uBLR) was run on all variables, with an outcome expressed by the beta coefficient, which describes the size and the direction of the relationship between predictor and the response variable (in this case in-hospital mortality), *p* value and odds ratio (OR) with its 95% confidence interval (CI). Continuous variables with significant differences assessed by uBLR were tested for their ability to discriminate between event and no-event by means of the ROC curve [plot of sensitivity vs (1 − specificity)]. Only variables for which the area under the curve (AUC) was ≥ 0.80, corresponding to good discrimination (AUC = 0.50 corresponds to chance) were dichotomized, setting the threshold at the value which satisfied the triple condition of: (1) maximization of the harmonic mean of sensitivity (SNS) and specificity (SPC), (2) maximation of Youden’s index (SNS + SPSC-1), and (3) minimization of the distance of the curve from the upper left corner (SNS = SPC = 1).

The dichotomous and dichotomized variables determined significant by the univariate analysis were then analyzed with a multivariate binary logistic regression (mBLR) to adjust for correlations between variables and extract the independent significant predictors of in-hospital mortality.

Correlation between variables was studied with the non-parametric Spearman correlation coefficient rho (range − 1:1).

Significance corresponded to *p* < 0.05 and 95% CI for RR and OR not including 1; 95% CI > 1 and beta > 0 indicated risk of the event; whereas, 95% CI < 1 and beta < 0 indicated protection.

The analysis was run on StatPlus: Mac v.7 (AnalysisSoft.Walnut.CA,USA).

## Results

The study sample of 346 patients was subdivided in two groups according to their final prognosis: patients who died during hospitalization (*n* = 83) and patients discharged after recovery (*n* = 263).

The baseline characteristics, comorbidities, clinical, laboratory and radiological data of the two groups are reported in Table 1 (Comorbidity, clinical, laboratory and radiological data of death vs. recovery COVID-19 patients)**,** subdivided for major clarity in four sections: I_Comorbidities, II_Clinical data, III_Laboratory data and IV_CRX. The results of the uBLR evidenced several significant differences between the two groups.

**Table 1:** Comorbidity, clinical, laboratory and radiological data of death vs. recovery COVID-19 patients

	Dead	Recovered	uBLR		
	(*n* = 83)	(*n* = 263)	Beta	*p* value	OR (95% CI)
Section I. Comorbidities					
Age (years old)	79 ± 11	60 ± 15	0.11	< 0.0001*	
Age > 75 years	56 (67%)	42 (16%)	2.40	< *0.0001*	10.9 (6.2–19.2)
Sex (male)	49 (59%)	159 (60%)	− 0.06	0.82	0.94 (0.57–1.56)
Hypertension	51/82 (62%)	96/251 (38%)	0.95	*0.0002*	2.6 (1.6–4.3)
Diabetes	17/79 (22%)	28/238 (12%)	0.72	*0.03*	2.1 (1.1–4.0)
Obesity	10/76 (13%)	17/241 (7%)	0.69	0.1	2.0 (0.9–4.6)
Smokers	18/80 (23%)	33/250 (13%)	0.65	0.05	1.9 (1.0–3.6)
Current smoker	7/39 (18%)	10/158 (6%)	1.20	*0.03*	3.2 (1.1–9.1)
Ex-smoker	9/39 (23%)	18/158 (11%)	0.84	0.06	2.3 (0.95–5.7)
Oncologic history	25/80 (31%)	32/263 (12%)	1.20	*0.0001*	3.4 (1.8–6.2)
FANS	3/60 (5%)	6/219 (3%)	0.63	0.39	1.9 (0.5–7.7)
ACEi	7/61 (11%)	28/223 (13%)	− 0.10	0.82	0.9 (0.4–2.2)
Sartans	11/55 (20%)	29/198 (15%)	0.40	0.31	1.5 (0.7–3.2)
Section II. Clinical data					
Onset of symptoms—CXR (days)	3 (2–6.5)	6 (2–8)	− 0.14	0.0005*	0.9 (0.8–0.94)
Fever	70/80 (88%)	236/254 (93%)	− 0.63	0.13	0.5 (0.2–1.2)
Cough	32/80 (40%)	171/255 (67%)	− 1.13	< *0.0001*	0.3 (0.2–0.5)
Rhinitis	1/79 (1%)	6/254 (2%)	− 0.63	0.56	0.5 (0.1–4.5)
Dyspnea	40/80 (50%)	94/254 (37%)	0.53	*0.04*	1.7 (1.0–2.8)
Pharyngodynia	1/79 (1%)	22/254 (9%)	− 2.00	0.05	0.14 (0.02–0.99)
Myalgias	2/79 (2%)	30/254 (30%)	− 1.60	*0.03*	0.19 (0.04–0.83)
Asthenia	6/79 (8%)	30/254 (12%)	0.49	0.3	0.61 (0.2–1.5)
Conjunctivitis	0/79 (0%)	3/254 (1%)	− 14.00	0.99	Nd
Anosmia	0/72 (0%)	4/248 (2%)	− 14.00	0.98	Nd
Dysgeusia	0/72 (0%)	9/248 (4%)	− 15.00	0.99	Nd
Headache	1/79 (1%)	13/254 (5%)	− 1.40	0.17	0.24 (0.03–1.85)
Nausea	2/79 (2%)	13/254 (5%)	− 0.73	0.34	0.5 (0.1–2.2)
Vomit	0/79 (0%)	9/254 (4%)	− 15.00	0.99	Nd
Diarrhea	5/79 (6%)	35/254 (14%)	− 0.86	0.08	0.42 (0.16–1.12)
Section III. Laboratory data					
WBC count (10^9^/L)	8.2 (5.3–12.6)	5.9 (4.6–7.9)	0.07	0.008*	1.07 (1.02–1.13)
WBC > 10 × 10^9^/L	33/74 (45%)	15/224 (7%)	2.40	< *0.0001*	9.2 (2.6–3.2)
Nr. of neutrophils (10^9^/L)	6.2 (3.6–9.5)	4.3 (3.0–5.8)	0.07	0.015*	1.1 (1.01–1.13)
% of neutrophils	81 (72–86)	72 (61–79)	1.94	0.05	6.9 (0.99–4.8)
Nr. of lymphocytes (10^9^/L)	0.9 (0.7–1.3)	1.1 (0.8–1.5)	0.01	0.62	1 (0.97–1.05)
% of lymphocytes	13 (8–20)	19 (13–27)	− 2.10	0.08	0.12 (0.01–1.3)
Neutrophils to lymphocytes ratio	6 (3–10)	3 (2–5)	0.37	< 0.0001*	1.4 (1.3–1.6)
Neutrophils to lymphocytes ratio > 4	48/65 (74%)	55/140 (39%)	1.80	< *0.0001*	6.1 (3.4–11.0)
Procalcitonin (ng/mL)	0.31 (0.14–0.81)	0.09 (0.05–0.14)	0.75	0.001*	2.1 (1.3–3.4)
Procalcitonin > 0.20 ng/mL	41/65 (63%)	24/143 (17%)	2.30	< *0.0001*	10.2 (5.5–19.0)
CRP (mg/L)	118 (64–185)	31 (10–92)	0.03	< 0.0001*	1.03 (1.02–1.03)
CRP > 60 mg/L	59/76 (78%)	83/234 (35%)	1.60	< *0.0001*	4.9 (2.9–8.3)
LDH (UI/L)	487.5 (383–745)	342 (283–422.5)	0.002	0.004*	1.002 (1.00–1.004)
LDH > 400UI/L	43/63 (68%)	37/116(32%)	1.15	*0.001*	3.2 (1.6–6.3)
Alteration hepatic values	43/75 (57%)	72/222 (32%)	1.05	< *0.0001*	2.9 (1.7–4.9)
CK elevation	20/42 (49%)	24/161 (15%)	1.70	< *0.0001*	5.4 (2.5–11.4)
PH	7.46 (7.42–7.49)	7.46 (7.44–7.49)	− 5.10	*0.04*	0.006 (0.00–0.84)
pO_2_ (mmHg)	59 (48–67)	70 (61–82)	− 0.04	0.0002*	0.96 (0.94–0.98)
pO_2_ < 65 (mmHg)	49/69 (71%)	59/164(36%)	1.45	< *0.0001*	4.3 (2.3–8.3)
pCO_2_ (mmHg)	34 (31–38)	34 (31–37)	0.03	0.14	1.03 (0.99–1.08)
Alveolar-arterial O_2_ gradient (mmHg)	74 (50–269)	45 (35–59)	0.01	< 0.0001*	1.006 (1.003–1.008)
Alveolar-arterial O_2_ gradient > 50 mmHg	48/65 (74%)	55/140 (39%)	1.50	< *0.0001*	4.4 (2.3–8.3)
PaO_2_/FiO_2_ ratio	222 (131–285)	309.5 (268–352)	− 0.01	< 0.0001*	0.99 (0.98–0.99)
PaO_2_/FiO_2_ ratio < 250	41/62 (66%)	24/143 (17%)	2.10	< *0.0001*	8.6 (4.4–17)
Section IV. CXR					
CXR simplified score	6 (4–6)	2 (0–5)	0.52	< 0.0001*	1.78 (1.45–1.95)
Brixia score	11 (7–14)	3(0–8)	0.23	< 0.0001*	1.26 (1.2–1.3)
Brixia score > 7	60 (72%)	71 (27%)	1.36	< *0.0001*	3.9 (2.3–6.5)
Pleural effusion	7 (8%)	16 (6%)	0.30	0.55	1.3(0.5–3.6)
Volume loss	7 (8%)	11 (4%)	0.81	0.15	2.2(0.8–6.1)

Significant differences were reported in italics

*uBLR* univariate binary logistic regression, *CXR* chest X-ray, *WBC* white blood cells, *CRP* C-reactive protein, *LDH* lactate dehydrogenase, *CK* creatine kinase

*Continuous variables

**Fig. 2:**
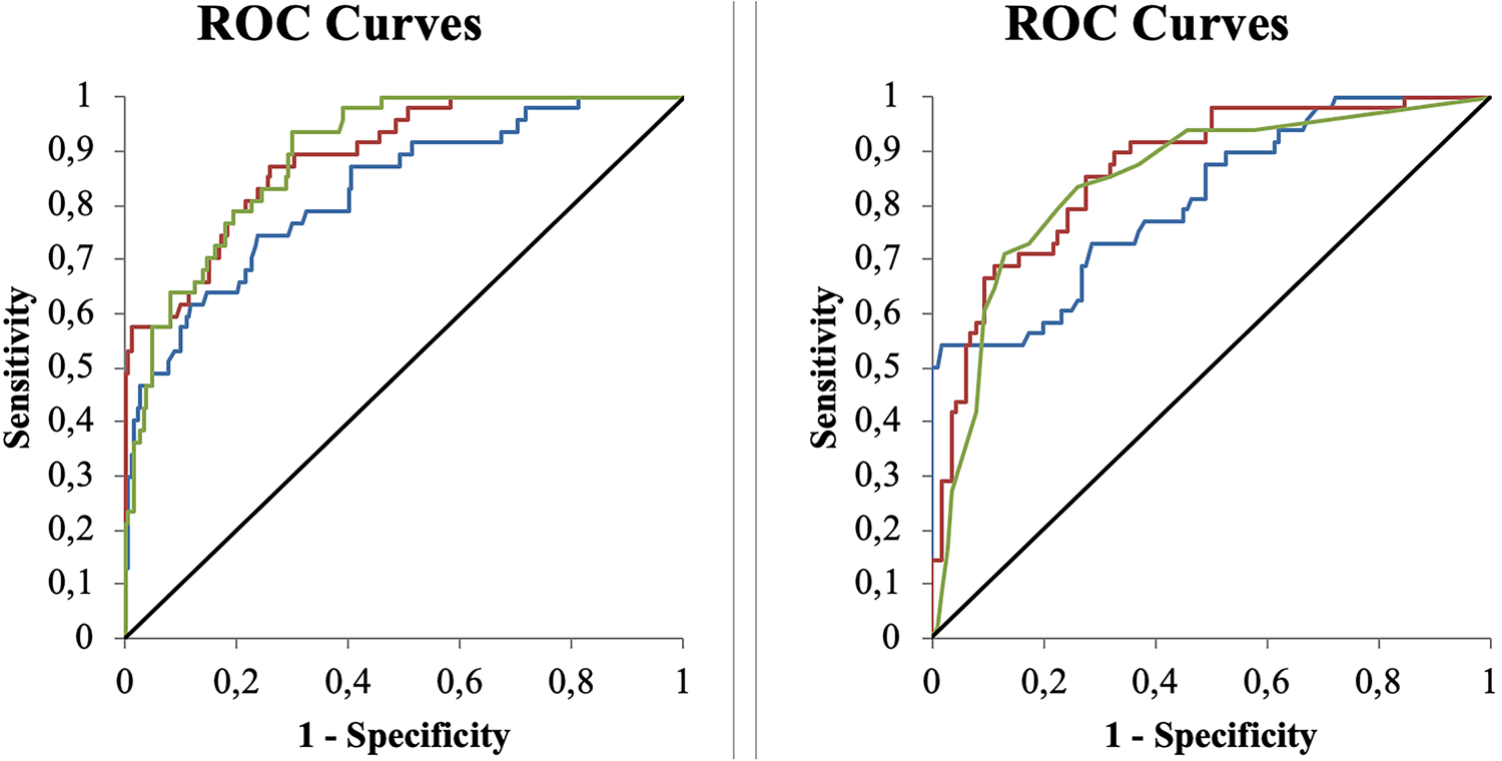
ROC curves. Left panel: age (red, AUC = 0.86); Brixia Score (green, AUC = 0.84), LDH (blue, AUC = 0.81). Right panel: CPR (red, AUC = 0.89), NLR (green, AUC = 0.89), PaO_2_/FiO_2_ (blue, AUC = 0.82) (color figure online)

The continuous variables with significant differences were tested for their ability to discriminate between in-hospital death and recovery by computing their ROC curve. Age, CXR simplified score, CXR Brixia score, P/F, A–a gradient, NLR, CRP, procalcitonin, WBC, PO_2_ and LDH, had AUC values between 0.75 and 0.90 (see Fig. 2) and were thus dichotomized. Both X-ray scores were significantly different between patients with good and bad prognosis (*p* < 0.0001), but the “Brixia score” had a slightly greater discriminating capacity than the simplified score (AUC = 0.81 vs. 0.79, *p* = 0.046). The dichotomization procedure determined as regions associated with significantly increased risk of in-hospital mortality: age > 75 years, Brixia score > 7, P/F < 250, A–a gradient > 50 mmHg, NLR > 4, CRP > 60 mg/L, procalcitonin > 0.20 ng/mL, WBC > 10 × 10^9^/L, PO_2_ < 65 mmHg and LDH ≥ 400 UI/L. The results on the dichotomized variable are reported in Table 1 in the line under the correspondent continuous one.

The mBLR run on the ensemble of dichotomous or dichotomized variables determined significant by univariate analysis yielded four independent significant predictors for in-hospital mortality: age > 75 years, Brixia > 7, P/F < 250 and CRP > 60 mg/L. Their number was limited by the intricate pattern of correlations among the variables of Table 1. As examples, age > 75 is directly correlated with hypertension (rho = 0.24), diabetes (0.18), oncological history (0.28), A–a gradient > 50 mmHg (0.24) and PO_2_ < 65 mmHg (0.30); Brixia > 7 is correlated with LDH > 400 (0.39), procalcitonin > 0.20 ng/mL (0.30) and NLR > 4 (0.30); CRP > 60 is correlated with PO_2_ < 65 mmHg (0.46) and LDH > 400UI/L (0.44); finally, P/F < 250 correlates with A–a gradient > 50 mmHg (0.60) and with PO_2_ < 65 mmHg(0.26). The main parameters of the mBLR outcome for the four independent predictors and two protectors are reported in Table 2 (outcome of multivariate binary logistic regression: predictors and protectors of in-hospital mortality) and show that age > 75 years was the factor most heavily associated with in-hospital mortality.

**Table 2:** Outcome of multivariate binary logistic regression: predictors (beta coefficient > 0) and protectors (beta coefficient < 0) of in-hospital mortality

Variable	Beta coefficient	*p* value	Odds ratio (95% CI)
Age > 75 years	2.8	< 0.0001	16 (6–43)
Brixia score > 7	1.05	0.03	2.9 (1.1–7)
CRP > 60 mg/L	1.3	0.01	3.6 (1.35–9.5)
PaO_2_/FiO_2_ ratio < 250	1.9	< 0.0001	7 (2.7–18)
Onset of symptoms	− 0.12	0.004	0.89 (0.82–0.96)
Cough	− 1.2	< 0.0001	0.29 (0.16–0.51)

**Fig. 3:**
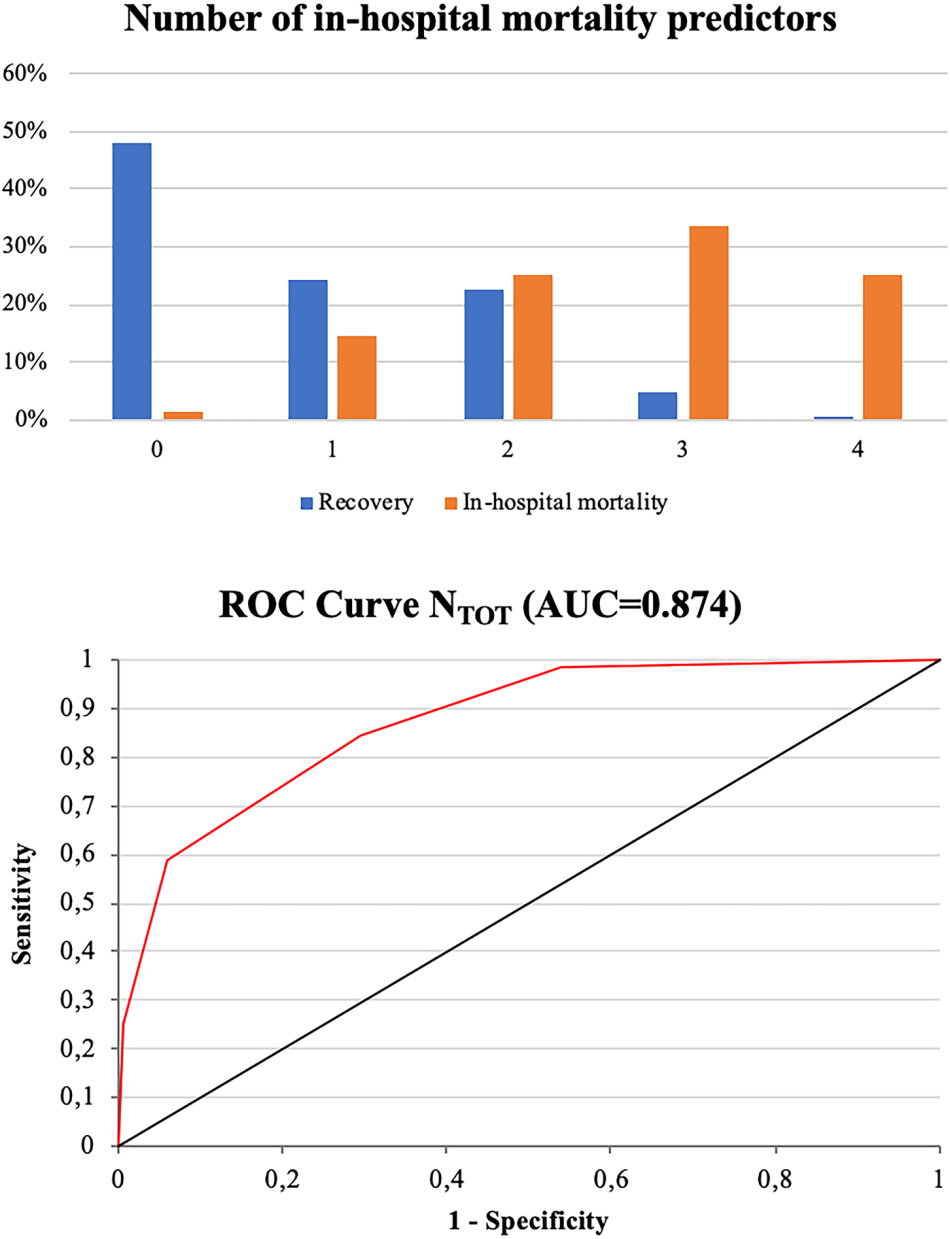
Number of in-hospital mortality predictors for each patient. Top panel: distribution of the total number *n* of risk predictors between dead and recovered patients. Bottom panel: ROC curve testing the discriminating ability of *n*

The total number of independent prognostic factors diagnosed for each patient was *N*_TOT_ = 1(0–2). The distributions of *N*_TOT_ for the patients who met in-hospital mortality and for the recovered patients are displayed and compared in Fig. 3, top panel (*p* < 0.0001). The ROC curve testing the discriminating ability of *N*_TOT_ is shown in Fig. 3, bottom panel: AUC = 0.87, with threshold for increased in-hospital mortality *N*_TOT_ ≥ 2. Among the patients with *N*_TOT_ < 2, the in-hospital mortality rate was 6% (no death for *N*_TOT_ = 0), against 58% for patients with *N*_TOT_ ≥ 2 (*p* < 0.0001; RR = 7.6(4.4–13). Actually 92% of patients who suffered in-hospital mortality arrived at the ER with a poor prognostic scenario including at least two out of the four independent negative predictors.

Age > 75 years was accompanied by *N*_TOT_ = 2 (1–3) other predictors against 1(0–2) for age ≤ 75 years (*p* < 0.001): in particular, the older age patients were further aggravated by Brixia > 7, P/F < 250 and CRP > 60 mg/L in 35% of cases against 10% for patients with minor seniority (*p* < 0.0001, RR = 3.5 (1.9–6.4).

**Fig. 4:**
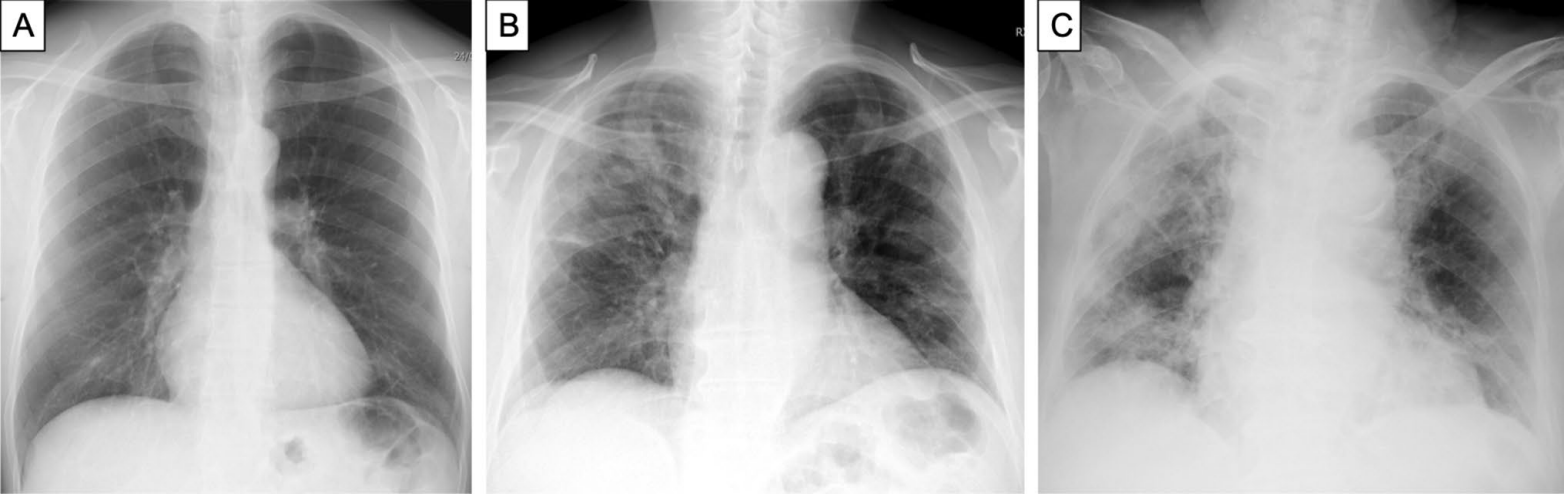
**a** 62-Year-old man who had the following values for the risk predictors when admitted to the emergency room: P/F = 417, CRP = 25.6 mg/L and Brixia score 2. Overall, the patient had no risk factor and he was discharged after recovery without the need of intensive care unit. **b** 67-Year-old man who had the following values: P/F = 314, CRP = 92.3 mg/L and Brixia score 7. Overall, the patient had one risk factor and was discharged after discharged after need of the intensive care unit. **c** 87-Year-old female who had the following values: P/F = 262, CRP = 91.2 mg/L and Brixia score 13. Overall, the patients had three risk factors and died during hospitalization

The impact of an increasing number of predictors N_TOT_ is illustrated in Fig. 4, which compares the CXR of patient A without predictors with those of patient B, with one predictor, and patient C with three predictors, including the most powerful, i.e. age > 75 years.

As final information, mBLR determined a short time lapse between onset of symptoms and CRX and coughing as significant protective factors (beta coefficient < 0): their figures are reported in the two bottom lines of Table 2.

## Discussion

Our study investigated the relationship of the set of demographics, clinical, laboratory and CXR data of 346 patients with COVID-19 as collected at their admission to the ER with their final prognosis (83 dead and 263 recovered). The intra-hospital mortality rate of 23% is in line with a recent meta-analysis that analyzed 60 studies and 51,225 hospitalized patients finding an in-hospital mortality rate of 24.3% [19].

Multivariate analysis determined the existence of four independent predictors for in-hospital mortality: age > 75 years, CRP > 60 mg/L, P/F < 250 and CXR “Brixia score” > 7. All these predictors had significant direct correlations with several other variables with prognostic value at the univariate analysis. The key finding was that a patient with a number of prognostic predictors *N*_TOT_ ≥ 2 had an eightfold higher risk of in-hospital mortality than patients with a minor number of predictors (58% vs 6%, *p* < 0.0001).

The result on age agrees with previous studies [5, 15, 20–22]. Older age is significantly associated with an increasing presence of several comorbidities such as diabetes, hypertension, oncological history, cardiovascular and kidney diseases, each of which tends to enhance the death risk. In particular, the age range > 75 years we determined associated with the worst prognosis is consistent with the data of Borghesi et al. [15], who identified in a population of the same ethnicity and with similar socio-demographic characteristics a cut-off of 71 years.

Among laboratory parameters, regression analysis showed elevated CRP values and low P/F (in our sample of patients, respectively, > 60 mg/L and < 250) as predictors for worse prognosis. An elevation of the concentration of CRP is often observed in COVID-19 patients. The study of Guan et al. [5] found that laboratory parameters that assessed inflammation and cell damage, such as CRP, were significantly higher in patients with a severe disease than in patients with a non-severe disease. CRP level significantly increases in COVID-19 patients due to inflammatory reaction and tissue destruction. High concentrations of CRP were reported to indicate more severe illness-correlated with lung damage and worse prognosis [21, 23].

The severity of respiratory failure appears to be the driving force behind the prognosis of COVID-19. Indeed, the clinical situation in the advanced stages of COVID19 is that of hypoxemic respiratory failure (hypo or hypercapnic depending on the phase and comorbidities). This results in severe arterial hypoxemia refractory to supplementary O_2_ therapy. The cause is the formation of intrapulmonary shunts as a result of interstitial involvement. This is also confirmed by our finding on the direct correlation between Brixia score and A–a gradient and the inverse correlation between Brixia score and P/F (Brixia vs A–a gradient rho = 0.27, vs P/F rho = − 0.36; Brixia > 7 vs P/F < 250, rho = 0.29). Our data confirmed P/F as an independent parameter for determining the severity and prognosis of acute respiratory distress syndrome [24]. The importance of P/F in these patients was recently stressed by Kishaba et al. [25], who demonstrated that serial monitoring of P/F was useful for predicting short-term prognosis in COVID-19 patients. [25]

Regarding radiological data, our study confirmed the usefulness of CXR classification as a prognostic factor, with a greater extension of lung involvement corresponding to a worse prognosis [14–16]. Toussie et al. [14] evaluated demographic, clinical and radiological data, without including laboratory data, and firstly demonstrated that baseline CXR, when using a lung zone severity score, could predict outcomes in young and middle-aged adults affected by COVID-19. In their study, the radiological score together with obesity resulted predictor of hospitalization, while only the radiologic score was predictor of hospitalization in intensive care; however, the impact of these data on the final outcome of the patients was not evaluated.

Another CXR score created for COVID-19 is the so-called Brixia score [15], first used for predicting the risk of intra-hospital mortality in a sample of 302 patients; Brixia score over 8 and age over 71 were associated with an increased risk of intra-hospital mortality, but without considering the laboratory data. In our study, we tested both scores and observed that the Brixia score, even if more complex, was more discriminating than the score proposed by Toussie et al. [14], and was therefore, chosen to be inserted among the predictors as Brixia score > 7.

Two of the variables analyzed appeared to be protective factors. First of all, a short time lapse between onset of symptoms and CXR theoretically correspond to an early diagnosis (since the CXR was made less than 24 h from RT-PCR) which is often synonymous with early treatment and was associated with a better prognosis in COVID19 patients: in particular, Huang et al. [26] found a positive correlation of the time from symptom onset to diagnosis and treatment and the time to disease resolution. Second, the presence of coughing, one of the most prevalent symptoms in COVID-19, resulted a protective factor, this data is in accordance with the recent meta-analysis of Mesas et al. [19] who reported an OR of 0.7 for COVID19 patient with cough.

This study has some limitations. The first is being a retrospective study on a relatively limited number of patients, even if it is one of largest in the literature dealing with the complex relation between clinical, laboratory, CXR and prognosis. The second is the lack of an external validation on different samples of patients.

In conclusion, our research indicates a set of early prognostic data for improving the COVID-19 pretreatment risk stratification. Even if the greatest risk of death from COVID-19 is aged above 75 years, elevated CXR score, high CRP levels and a low P/F at admission to the emergency department represent significant prognostic factors. A patient admitted to the emergency department with ≥ 2 predictors should immediately undergo intensive and aggressive treatments to reduce the risk of a more severe course of the disease, which may otherwise lead to an eightfold higher in-hospital mortality rate than for patients with a lower number of predictors.

## Abbreviations

COVID-19: Coronavirus disease 2019
SARS-CoV-2: Severe acute respiratory syndrome coronavirus 2
RT-PCR: Real-time reverse transcriptase-polymerase reaction chain test
CXR: Chest X-rays
WBC: White blood cellscount
NLR: Neutrophil to lymphocyte ratio
CRP: C-reactive protein
LDH: Lactate dehydrogenase
CK: Creatine kinase
PCT: Procalcitonin
PaO_2_: Arterial partial pressure of oxygen ()
PaCO_2_: Arterial partial pressure of carbon dioxide ().
FiO_2_: Fraction of inspired oxygen ()
A–a gradient: Alveolar–arterial gradient
P/F: PaO_2_/FiO_2_ ratio
BLR: Binary logistic regression
